# Design and Simulation of a Ratiometric SPR Sensor Based on a 2D van der Waals Heterojunction for Refractive Index Measurement

**DOI:** 10.3390/nano13030515

**Published:** 2023-01-27

**Authors:** Jun Zhou, Xiantong Yu, Lianzhen Zhang, Xuejing Liu, Youjun Zeng, Xuedian Zhang

**Affiliations:** 1 Key Laboratory of Optical Technology and Instrument for Medicine, Ministry of Education, University of Shanghai for Science and Technology, Shanghai 200093, China; 2 School of Physics & Optoelectronic Engineering, Guangdong University of Technology, Guangzhou 510006, China

**Keywords:** SPR sensors, strong coupling, two-dimensional van der Waals heterojunction

## Abstract

Surface plasmon resonance (SPR) sensors have been widely applied in many fields because of their advantages of working in real time and high sensitivity. However, because the spectrum of an SPR sensor is easily affected by the smoothness of the metal surface, this type of sensor has obvious disadvantages in the application of quantitative detection. We designed an SPR refractive index sensor for molecular detection that has the advantage of quantifiability. A ratio spectral quantitative analysis method was established based on the two coherent dips of the SPR spectrum formed by the strong coupling effect between the surface plasmon polaritons and the excitons of the J-aggregate molecule 5,6-dichloro-2–[3–[5,6-dichloro-1-ethyl-3–(4-sulfobutyl)–2-benzimidazoline subunit] propenyl]–3-ethyl-1–(4-sulfobutyl) benzimidazole hydroxide inner salt (TDBC). The introduced MoS_2_/graphene van der Waals heterojunction produced an effective charge transfer to the Ag film, resulting in significant electric field enhancement at the sensing interface and further improving the detection sensitivity of the sensor. The simulation results showed that for 43 nm Ag film, for example, the ratiometric SPR sensor with the Ag film structure can obtain 16.12 RIU^−1^ sensing sensitivity, applied to the detection of gas molecules, while the SPR sensor with single-layer graphene and three layers of MoS_2_ heterostructures can obtain 50.68 RIU^−1^ sensing sensitivity. The addition of van der Waals heterostructures can significantly improve sensing performance by 215%.

## 1. Introduction

SPR sensors based on the surface plasmon resonance (SPR) effect of noble metal materials have received extensive attention and garnered in-depth research in recent years [1,2]. The basic principle of an SPR sensor is that the SPR spectrum is closely related to the refractive index of the surface of the metal; that is, when the refractive index of the surface of the metal material changes very slightly, the position of the SPR resonance peak also changes accordingly [3,4]. Sensitive detection technology based on this principle is widely used in various fields and has the advantages of working in real time and having high efficiency and sensitivity [3,5].

However, some disadvantages of using SPR sensing devices in practical applications have gradually become apparent. The most notable problem is the defect of SPR sensors in quantitative analysis. Due to the dependence of SPR sensors on noble metal materials and the fluctuation of the surface uniformity of noble metal nanostructures, the local electric field has an uneven distribution, resulting in irregular fluctuation of the spectral intensity and peak position, which makes quantitative analysis difficult and significantly restricts further promotion of the sensitive detection technology of SPR [6]. At present, there are mainly two ways to solve this. One method is to establish a mathematical statistical model with a large amount of spectral data to reduce the impact of this fluctuation on the results to a certain extent [7]. Another method is to use a labeled molecule as a reference to establish a ratio spectral analysis method to eliminate the influence of the instrument and the sensor [8]. These two ideas partly solve the quantitative analysis problem of SPR sensors and broaden their application prospects. The advantage of the mathematical statistical method is that it has no additional sample preparation process, and the operation is simple. The disadvantage is that a large amount of data must be obtained, which requires more time and higher costs. The method of labeled ratio spectral analysis increases the workload in the sample preparation process, and the fundamental problem is finding suitable label molecules. Consequently, it is necessary to develop and design a label-free quantitative analysis method.

Van der Waals heterojunctions (vdWHs) are new composite two-dimensional (2D) materials with different 2D materials stacked together using van der Waals force [9]. The interaction intensity of van der Waals force is generally smaller than that of chemical bonds (approximately 0.1–10 kJ/mol) [10]. The van der Waals integration strategy can be applied to any material, especially for the convenient integration of materials with different band structures and electronic properties [11]. At present, many methods can be used to prepare vdWHs, including mechanical stripping and aligned transfer [12], physical vapor deposition/transport growth (PVD) [13], and chemical vapor deposition (CVD) [14]. Each method has advantages. Generally speaking, vdWHs prepared by mechanical stripping and aligned transfer have the advantages of fewer defects, smoothness, and high mobility. However, the yield of this method is low, and the sample is easily contaminated. The PVD method applies to a wide range of materials and is easy to operate, but the interface is easily disturbed by defects. The quality of vdWHs produced using the CVD method is the best, but the transfer process is easily polluted, and the operation process is complex [15]. These vdWHs are some of the best potential candidate materials for the design of novel optoelectronic devices. Relevant studies have found that vdWHs have strong charge transfers due to the existence of the interlayer Fermi energy level difference. Therefore, it is of great significance to use vdWHs to improve the sensitivity of SPR sensors and to design new sensors [16,17,18]. In fact, recent reports on sensitive SPR detection technology based on vdWHs have confirmed this assumption [19,20]. Therefore, we introduced vdWHs to increase detection sensitivity, which is expected to broaden the application of SPR sensors in quantitative analysis.

In this study, based on the performance of the curves of the two dips originating from the strong coupling effect between surface plasmon polaritons (SPPs) and molecular excitons [21,22,23,24], we proposed a novel ratio quantitative detection technology. Additionally, we used vdWHs to increase detection sensitivity.

## 2. Calculation Model and Method

**Figure 1:**
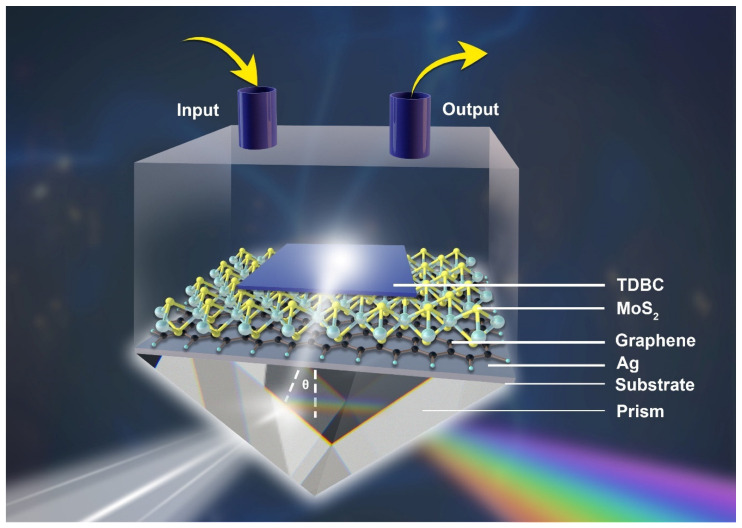
Diagrammatic sketch of the SPR sensor based on van der Waals heterojunction sensitization.

The sensing area of the SPR sensor in this study is a five-layer system that is based on vdWHs and consists of glass, silver film, graphene, molybdenum disulfide (MoS_2_), and polyvinyl alcohol (PVA) mixed with TDBC film. A diagrammatic sketch is shown in Figure 1. The sample chamber was designed to achieve circulation of the air or liquid environment. The input and output can be respectively connected with a microfluidic pump to achieve the flow of the target detector and improve the accuracy of detection. The organic molecule TDBC is a J-aggregation molecule. When the TDBC molecules in the PVA solution reach a certain concentration, they undergo J-aggregation. The wavelength range of the detection light is 475 nm–960 nm [25]. PVA is a porous medium, so small molecules can pass through the pores and adhere to the surface of the detection layer through electrostatic adsorption. The change in the number of molecules near the surface of the sensing layer causes a small change in the refractive index near the interface. The small refractive index change in the sensing interface can lead to a change in the SPR signal. Therefore, the change in the refractive index of the sensing medium surface can be determined by monitoring the change in the SPR signal to quantitatively analyze the target detection object.

Before examining the detection performance of this sensor, the optical parameters of the SPR sensor should be determined. In this work, the material of the prism was SF11 glass, and the substrate was BK7 glass. The refractive indexes of the above materials were obtained from a reference source [26].

Using the Drude–Lorentz model [18], the complex refractive index of the silver film can be obtained, as shown in Formula (1):

(1) $$n_{silver}={\left(\varepsilon_{r}+i\varepsilon_{i}\right)}^{\frac{1}{2}}={\left(1-\frac{\lambda^{2}\lambda_{c}}{\lambda_{p}^{2}\left(\lambda_{c}+i\lambda \right)}\right)}^{1/2}$$

Parameters $\lambda_{p}$ and $\lambda_{c}$ are the plasma wavelength and collision wavelength of silver, respectively.

The complex refractive index of single-layer graphene is fitted from the experimental data. The thickness of the graphene layer is $d_{graphene}=L_{1}\times 0.34\;\mathrm{nm}$, with *L_1_* representing the layer number of graphene [26]. According to the experimental data, the complex refractive index of the 2H phase MoS_2_ layer is obtained by fitting [27] (see refractive index calculation section, Part B, supporting information), which makes use of its semiconductor characteristics to form a van der Waals heterostructure with graphene to achieve significant improvement in sensor sensitivity in the wavelength range of 400 nm–1100 nm. The thickness of the MoS_2_ layer is $\;d_{MoS_{2}}=L_{2}\times 0.65\;\mathrm{nm}$, where *L_2_* is the number of MoS_2_ layers, and the thickness of the single-layer MoS_2_ is approximately 0.65 nm. The refractive index of the medium to be measured is given by the following relationship:

(2) $$n_{sample}=1+∆n$$

∆n represents the small refractive index change caused by binding of the target analyte.

The J-aggregate molecule 5,6-dichloro-2–[3–[5,6-dichloro-1-ethyl-3–(4-sulfobutyl)–2-benzimidazoline subunit] propenyl]–3-ethyl-1–(4-sulfobutyl) benzimidazole hydroxide inner salt (CAS No.: 10049–96-4) is abbreviated as TDBC. The absorption peak of the J-aggregation of TDBC is 590 nm [25].

In the sensing layer designed in this study, TDBC molecules were mixed in PVA film. In the simulation calculation, the complex dielectric constant of the PVA/TDBC layer is as follows [28]:

(3) $$\varepsilon_{layer}\left(e\right)=\varepsilon_{PVA}\left(e\right)+\frac{f{\left(\hbar q\right)}^{2}}{m\varepsilon_{0}L_{z}\left(e_{0}^{2}-e^{2}-i\gamma_{0}e\right)}$$

$\varepsilon_{PVA}\left(e\right)\;$ is the dielectric constant of PVA, *f* is the oscillation intensity of J aggregates, $\gamma_{0}$ is the line width of the exciton, $e_{0}$ is electron energy, *q* is the amount of electron charge, and m is the mass of electrons. $\varepsilon_{0}$ is the dielectric constant in free space.

(4) $$\varepsilon_{PVA}\left(e\right)=1.51162+\frac{46399}{\lambda^{2}}-4.32127\times \frac{10^{9}}{\lambda^{4}}$$

$f=1.8\times 10^{18}$ *,*

$\gamma_{0}=49\;mev$ *,*

$e_{0}=2100\;mev$ *,*

$q=1.602176487\times 10^{-19}$ *,*

$\hbar =1.05457162\times 10^{-34}\times 6.24150974\times 10^{21}$ *,*

$L_{z}=25\;nm$ *,*

$m=9.10938215\times 10^{-12}$.

Reflectivity changes in the SPR sensing system were studied using the N-layer film transfer matrix method (TMM) and the Fresnel equation [29,30,31] (Part A in Supplementary Materials). In the proposed model, it is assumed that all of the layered films are stacked parallel to the Ag film. All of the stacked layers are considered optically isotropic and nonmagnetic.

The detection sensitivity *S* and enhancement factor *EF* are used to evaluate the detection performance of the proposed SPR sensor. For example, the detection sensitivity *S* of the vdWH/Ag film structure $S_{vdWhs/Ag}\;$ is determined with Equation (5):

(5) $$S_{vdWhs/Ag}=\frac{∆r}{∆n}$$

Δr represents the change value of *I_A_/I_B_* within the unit range (*I_A_* and *I_B_* are the intensity integrals of the two dips caused by strong coupling). Compared with the detection sensitivities of the Ag/vdWHs and the Ag film SPR sensor, the enhancement factor *EF* can be calculated using the following formula:

(6) $$EF=\frac{S_{vdWhs/Ag}-S_{Ag}}{S_{Ag}}\times 100\%$$

## 3. Results and Discussion

### 3.1. Strong Coupling Effect in the Ag/vdWHs/TDBC System

**Figure 2:**
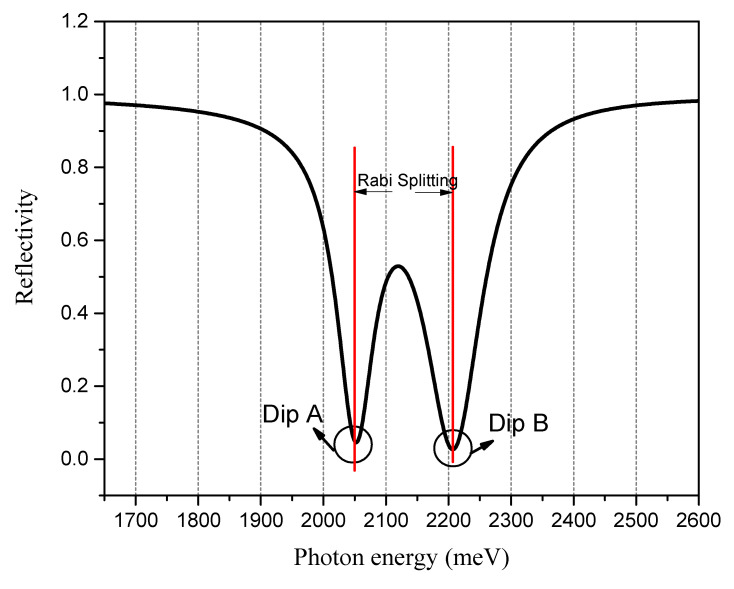
The strong coupling effect between SPP and TDBC excitons. The thickness of the Ag film is 42 nm, and the incident angle of excitation light is 49°.

Reflectivity (RP) is the function of the incident wavelength (λ), which is called the SPR reflection curve or SPR reflection spectrum. The SPR resonance wavelength corresponds to the incident light wavelength at the minimum reflectivity of the curve. When the strong coupling effect occurs, the SPR reflection spectrum shows the characteristics of the two minimum reflectivities (shown in Figure 2), and the difference between the two minimum reflectivities is called Rabi splitting. The value of the two minimum reflectivities is adjusted by changing the refractive index near the sensing surface, so it is used in the design of the ratio SPR sensor. First, it is necessary to determine the influence of the structural parameters of the designed model on the strong coupling effect. The refractive index of the medium to be measured is fixed at 1.000, corresponding to the refractive index of air.

**Figure 3:**
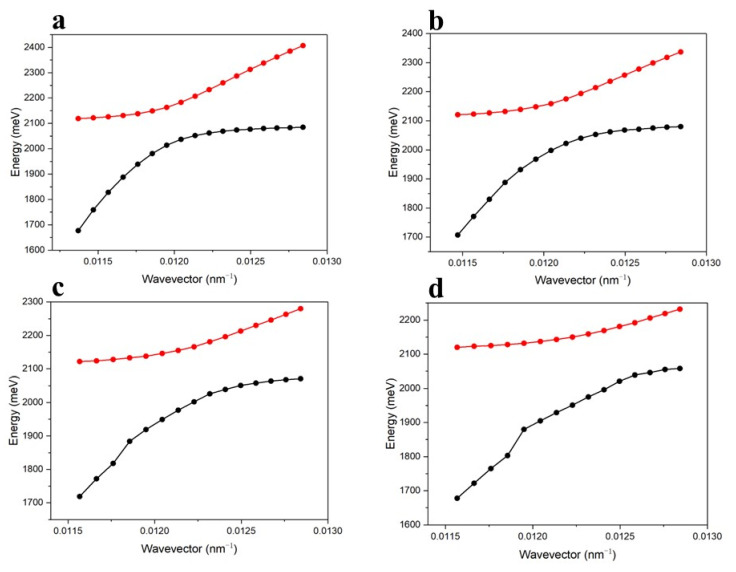
The strong coupling effect regulated by the thickness of the MoS_2_ layers. The thickness of the Ag film is 42 nm, and the thickness of the graphene is a monolayer. (**a**–**d**): The number of MoS_2_ layers is 0–3. The red and black dotted lines, respectively, represent the upper and lower energy branches produced by the strong coupling effect of the polaron–exciton system.

We also focused on the influence that the thickness of the vdWHs has on the strong coupling effect of the designed SPR sensor. As shown in Figure 3, by altering the thickness of the Ag film and MoS_2_, the variation in the positions of the two dips of the proposed SPR sensor with the wave vector is $k=\frac{2\pi }{\lambda }*n_{c}*sin\left(\theta \right)$, *n_c_* is the prism refractive index at the wavelength of *λ*, and *θ* is the incident angle [28]. For the Ag film with fixed thickness, the two dips have an obvious red shift with the increase in the number of MoS_2_ layers, and the Rabi splitting value becomes larger, which is due to the electron energy loss with an increase in the thickness of the MoS_2_ layer. Taking Figure 3d as an example, when MoS_2_ is a three-layer structure, we can control the SPR resonance condition by changing the incident angle of light in order to study the strong coupling effect between SPP and TDBC. The red and black points represent the positions of the two dips when the strong coupling effect occurs in this system. By comparing the Figure 3a–d diagram, the results show that the number of layers of MoS_2_ changes the value of the Rabi splitting. When the thickness of the Ag film is 42 nm and the number of layers of MoS_2_ are 0, 1, 2, and 3, the Rabi splitting intensity changes of the strong coupling effect in the above system are 150 meV, 151 meV, 164 meV, and 199 meV, respectively.

**Figure 4:**
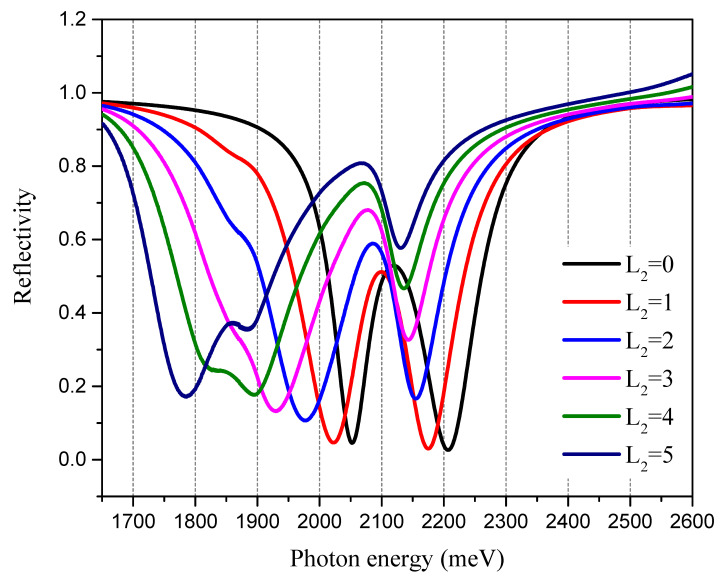
The strong coupling effect between the SPPs and the TDBC exciton regulated by the MoS_2_ thickness. The thickness of the Ag film is 42 nm, the incident angle of the excitation light is 49°, and L2 is the number of MoS_2_ layers.

When the thickness of the Ag film is 42 nm and the incident angle of the excitation light is 49°, the SPR reflection spectrum changes as the number of MoS_2_ layers changes. The results show that as the MoS_2_ thickness increases, the linewidth of the two dips increases (Figure 4), which is due to greater electron energy loss caused by the increase in the thickness of the MoS_2_ layer.

### 3.2. Sensing Performance of the SPR Sensor

Next, the sensing performance of the proposed SPR sensor was evaluated. In specific detection performance, it is necessary to select the incident angle corresponding to the spectrum when the intensities of the two dips are similar. According to the results shown in Figure 3, we tested the SPR reflection spectrum at an incident angle of 49°. The Ag film thickness was 43 nm, the graphene thickness was a single layer, and the MoS_2_ thickness was three layers (Figure 4). The results show that there is little difference in the intensities of the two dips of the SPR spectrum, which is the basic requirement for the design of ratio spectral sensing.

**Figure 5:**
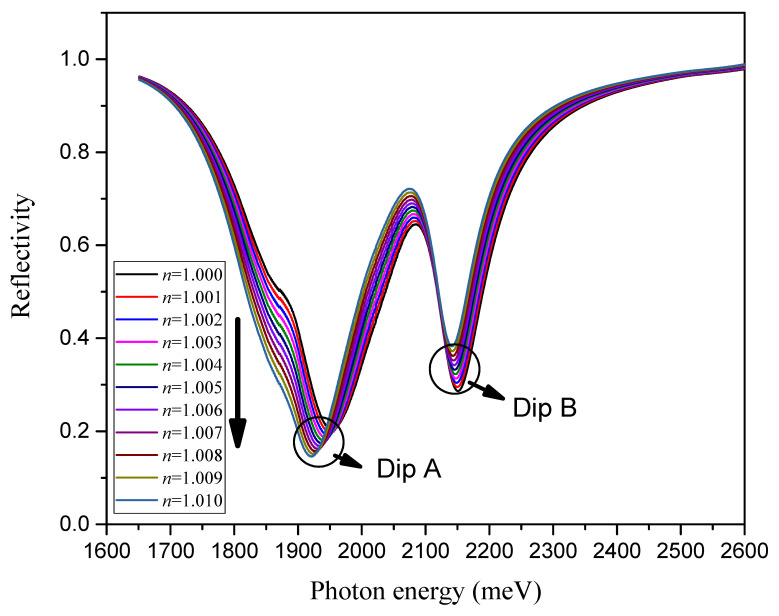
SPR reflection spectrum for a variable environmental refractive index with 43 nm Ag film, single-layer graphene, and three-layer MoS_2_.

The strong coupling effect between the SPR polaron and exciton is very sensitive to changes in the refractive index of the external environment. In fact, it can be seen in Figure 5 that the environmental refractive index has a different influence on the two dips of the SPR spectrum. There were significantly greater changes in the low-energy branch than in the high-energy branch.

**Figure 6:**
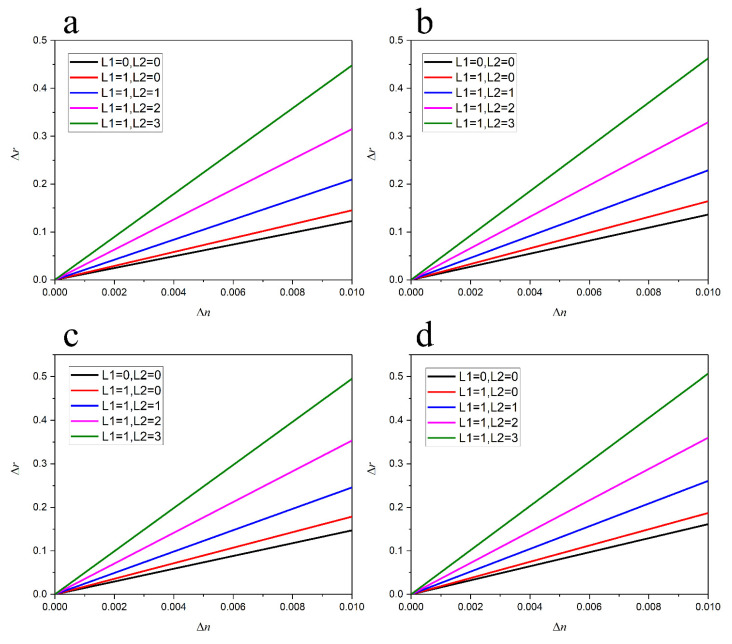
Detection sensitivity of the SPR sensor effect with MoS_2_ thickness. (**a**–**d**) show the sensing performance analysis of the SPR sensor with Ag film thicknesses of 40 nm, 41 nm, 42 nm, and 43 nm, respectively. L1 is the number of layers of graphene, and L2 is the number of layers of MoS_2_.

When the refractive index changes ∆n = 0.001, changes in the intensity ratio *I_A_/I_B_* of the two dips can be investigated by changing the thickness of the Ag film and the number of layers of MoS_2_. Figure 6a–d show the thickness of the Ag film for 40 nm, 41 nm, 42 nm, and 43 nm. The number of layers of MoS_2_ increases from zero to three, and the detection performance of the ratio of the SPR sensor improves. The results show that for an Ag film/TDBC sensor, as the thickness of the Ag film increases, the detection sensitivity reaches 16.1 RIU^−1^ with 43 nm Ag film (the red curve in Figure 6d).

The introduction of graphene can improve the detection sensitivity of the sensor. When the Ag film thickness is 43 nm, the detection sensitivity of the SPR sensor increases by 16% compared to that of single-layer graphene. The use of vdWHs further improves detection sensitivity.

First, we investigated the detection sensitivity of the SPR sensor with Ag films of varying thicknesses. When the Ag film thicknesses were 40 nm, 41 nm, 42 nm, and 43 nm, the results show that the detection sensitivity of the three layers of MoS_2_ deposited on the 40 nm Ag film is 44.8 RIU^−1^, that of the three layers of MoS_2_ deposited on the 41 nm Ag film is 46.3 RIU^−1^, that of the three layers of MoS_2_ deposited on the 42 nm Ag film is 49.5 RIU^−1^, and that of the three layers of MoS_2_ deposited on the 43 nm Ag film is 50.7 RIU^−1^. Thus, the detection sensitivity of the sensor increases as the Ag film thickness increases. When the refractive index of the medium changed in the range of 1.001–1.010, a detection sensitivity of 50.7 RIU^−1^ was obtained. This sensitivity is 171% higher than that of the single-layer graphene/Ag film composite structure and 215% higher than that of the Ag film structure (Figure 6).

Second, the results illustrated in Figure 6 also show that the detection sensitivity of the sensor was affected by MoS_2_ thickness. When the thickness of the Ag film was 42 nm, the thickness of the graphene was one layer, and the number of MoS_2_ layers changed from zero to three. The detection sensitivities of the sensor were 17.8 RIU^−1^, 24.5 RIU^−1^, 35.3 RIU^−1^, and 49.5 RIU^−1^, respectively. Thus, as the number of MoS_2_ layers increases, the detection sensitivity of the SPR sensor increases. Compared with the Ag film SPR sensor, the ratio SPR sensor based on a MoS_2_/graphene heterojunction obviously has higher detection sensitivity.

**Figure 7:**
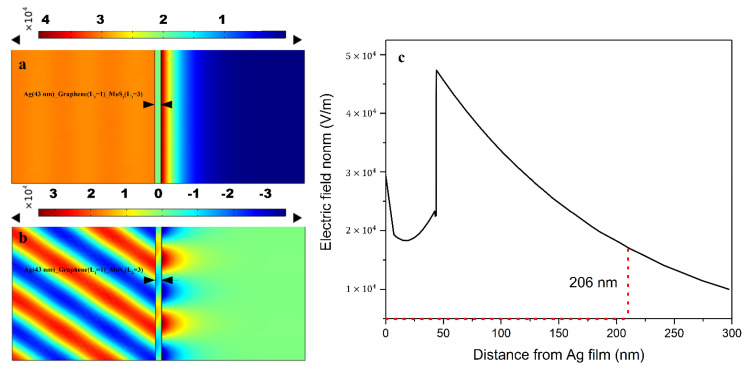
Electric field distribution (**a**) and *y* component (TM mode) (**b**) in 2D space based on a composite structure composed of single-layer graphene, three-layer MoS_2_, and 43 nm thick Ag film with SPR angle. (**c**) Evanescent decay of the excited electric field penetration into the sensing regime (the red line denotes that the electric field intensity attenuates to 1/e, ~206 nm).

In order to verify the SPR enhancement effect produced by the composite structure composed of single-layer graphene, three-layer MoS_2_, and 43 nm thick Ag film, the electric field distribution at the SPR resonance angle was simulated using the FEA method (Figure 7). The results show that a large electric field enhancement is generated at the sensing interface, with the intensity exponentially decaying into the sensing medium with 160.71 nm (the distance from the place with the maximum electric field strength to 1/e, i.e., 206 nm minus the thickness of the Ag film and vdWHs) penetration (i.e., the distance at which the electric field intensity decays to 1/e).

**Figure 8:**
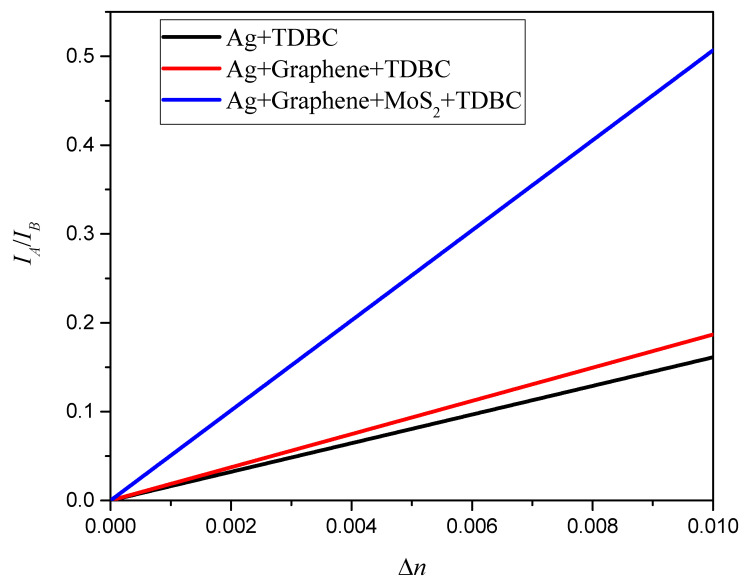
Sensing performance of three SPR sensing platforms: 43 nm thick Ag film (**black**), Ag film coated with single-layer graphene (**red**), and Ag film coated with single-layer graphene and three layers of MoS_2_ (**blue**).

The detection sensitivity of the SPR sensor was further measured according to the enhancement factor (EF). At present, the detection sensitivity of the proportional SPR refractive index sensor we designed is 50.68 RIU^−1^. Compared with other types of SPR sensors [18,19,24,26,29], the detection sensitivity of the proportional SPR refractive index sensor is not significantly better, but it is slightly better than that of the ordinary wavelength-type sensor. On the other hand, compared with the phase-type SPR sensor with high detection sensitivity, the detection sensitivity gap is large. The main advantage of the proportional SPR refractive index sensor designed in this study is that it can effectively eliminate the numerical jitter caused by material defects and instrument fluctuations by using the intensity ratio of the spectrum. Its application prospects mainly focus on quantitative analysis. The sensitivities of the three SPR sensing platforms are compared in Figure 8. The simulation results show that the proposed ratio SPR sensor, based on an Ag film/graphene/MoS_2_ structure, is very sensitive to changes in the refractive index. Under the same thickness of Ag film (43 nm), the detection sensitivity of the single-layer graphene/three-layer MoS_2_/Ag film SPR sensor is 171% higher than that of the single-layer graphene/Ag film SPR sensor and 215% higher than that of the Ag film SPR sensor.

## 4. Conclusions

In this research, a ratio SPR sensor for quantitative analysis based on the strong coupling effect between SPPs and excitons was designed. The use of vdWHs leads to a charge transfer between the graphene, MoS_2_, and Ag films, resulting in a significantly enhanced electric field near the interface, which effectively improves the detection sensitivity. By studying the influence elements of the detection sensitivity, we found that the detection sensitivity of 43 nm Ag film and three layers of MoS_2_ can reach 50.7 RIU^−1^. This sensitivity is 171% higher than that of a graphene/Ag composite nanostructured SPR sensor, which further demonstrates that vdWHs play a significant role in improving detection sensitivity. This sensor has the advantage of quantitative analysis for molecular detection. It is expected to play an important role in the case of detection that is difficult to label, such as the quantitative detection of gas molecules, in the future.

## Data Availability

Not applicable.

## Supplementary Materials

The following are available online at https://www.mdpi.com/article/10.3390/nano13030515/s1, Table S1: Refractive Index of MoS_2_.
