# Supplementary material for: Design and Simulation of a Ratiometric SPR Sensor Based on a 2D van der Waals Heterojunction for Refractive Index Measurement
Source: Nanomaterials (Basel). 2023 Jan 27;13(3):515. doi: 10.3390/nano13030515 (PMC9919535; doi:10.3390/nano13030515)
Supplement: Supplementary file 1 [file nanomaterials-13-00515-s001.zip › nanomaterials-2078975-supplementary.pdf]

Supplementary Materials

Design and Simulation of a Ratiometric SPR Sensor Based on a 2D van der Waals Heterojunction for Refractive Index Measurement

Jun Zhou [1], Xiantong Yu [1,*], Lianzhen Zhang [1], Xuejing Liu [1], Youjun Zeng [2] and Xuedian Zhang [1,*]

[1] Key Laboratory of Optical Technology and Instrument for Medicine, Ministry of Education, University of Shanghai for Science and Technology, Shanghai 200093, China
[2] School of Physics & Optoelectronic Engineering, Guangdong University of Technology, Guangzhou 510006, China
* Correspondence: xtyu@usst.edu.cn (X.Y.); xdzhang@usst.edu.cn (X.Z.)

Part A: Transfer matrix method（TMM）for reflection of multilayer films:

In this work, the reflection of multilayer films is measured by using the transfer matrix method（TMM）and Fresnel equation of multilayer film [1–3]. All layers in the system are considered to be optically isotropic and nonmagnetic.

Reflection coefficient, r_p, can be described by Fresnel equation and Snell's law:

$$r_p = \frac{E_{rp}}{E_{ip}} = \frac{n_t \cos\theta_i - n_i \cos\theta_t}{n_t \cos\theta_i + n_i \cos\theta_t} \quad \text{(S1)}$$

$$n_i \sin\theta_i = n_t \sin\theta_t \quad \text{(S2)}$$

n_i and n_t are the refractive indexes of the two media at the interface respectively.

For multi-layer case, the transfer matrix method is used for calculation. The matrix can be described as follows:

$$M = \prod_{k=2}^{N-1} M_k = \begin{bmatrix} M_{11} & M_{12} \\ M_{21} & M_{22} \end{bmatrix} \quad \text{(S3)}$$

$$M_k = \begin{bmatrix} \cos\beta_k & -i\frac{\sin\theta_k}{q_k} \\ -iq_k \sin\beta_k & \cos\beta_k \end{bmatrix} \quad \text{(S4)}$$

$$q_k = \frac{(\varepsilon_k - n_{SF11}^2 \sin^2\theta_1)^{1/2}}{\varepsilon_k} \quad \text{(S5)}$$

$$\beta_k = \frac{2\pi n_k \cos\theta_k (Z_k - Z_{k-1})}{\lambda} = \frac{\pi d_k}{2}(\varepsilon_k - n_1^2 \sin^2\theta_1)^{1/2} \quad \text{(S6)}$$

n_k is Refractive index of the *k*-th layer, θ_1 is the angle of incidence of the first layer, ε_k and d_k are respectively the dielectric function and thickness of the *k*-th layer。

The first boundary of the tangential field is defined as Z_1=0, last boundary Z_n=1:

$$\begin{bmatrix} U_1 \\ V_1 \end{bmatrix} = M \begin{bmatrix} U_{N-1} \\ V_{N-1} \end{bmatrix} \quad \text{(S7)}$$

where *U* is the tangential component of the interface electric field and *V* is the tangential component of the magnetic field. Therefore, for the *n*-th layer *p*-polarized light, the reflection coefficient is,

$$r_p = \frac{(M_{11} + M_{12}q_N)q_1 - (M_{21} + M_{22}q_N)}{(M_{11} + M_{12}q_N)q_1 + (M_{21} + M_{22}q_N)} \tag{S8}$$

And the reflection coefficient of SPR, R_P,

$$R_P = |r_p|^2 \tag{S9}$$

Equation (S8), (S9) is also applies to s-polarized light, and the parameters,

$$q_k = (\varepsilon_k - n_{SF11}^2 sin^2\theta_1)^{1/2} \tag{S10}$$

Part B: Refractive Index calculation of MoS_2

According to the experimental data [4], the complex refractive index of MoS_2 layer is obtained by fitting. The dielectric function of MoS_2 can be expressed by the following equation.

$$\varepsilon_{MoS_2} = \varepsilon_r + i\varepsilon_i \tag{S11}$$

ε_r and ε_i are respectively the real part and imaginary part of the dielectric function.

Table S1. Refractive Index of MoS_2.

wavelength	ε_r	ε_i	wavelength	ε_r	ε_i
500.0814	24.63127	10.81341	610.5496	22.42838	14.30749
500.7591	24.56813	10.78345	611.2273	22.61936	14.51316
501.4368	24.50449	10.75379	611.905	22.802	14.70985
502.1145	24.44036	10.72441	612.5827	22.97537	14.89655
502.7923	24.37575	10.69532	613.2605	23.13851	15.07224
503.47	24.31068	10.66652	613.9382	23.29048	15.2359
504.1477	24.24517	10.63801	614.6159	23.43033	15.38651
504.8254	24.17922	10.60978	615.2936	23.55714	15.52307
505.5031	24.11287	10.58183	615.9713	23.66994	15.64455
506.1809	24.04611	10.55417	616.6491	23.76781	15.74994
506.8586	23.97896	10.52679	617.3268	23.84978	15.83823
507.5363	23.91145	10.49969	618.0045	23.91493	15.90839
508.214	23.84358	10.47287	618.6822	23.96231	15.95941
508.8917	23.77537	10.44633	619.3599	23.99097	15.99027
509.5694	23.70683	10.42007	620.0377	24	15.99998
510.2472	23.63798	10.39409	620.7154	23.99858	15.9929
510.9249	23.56883	10.36838	621.3931	23.99469	15.97344
511.6026	23.49941	10.34295	622.0708	23.98843	15.94215
512.2803	23.42971	10.3178	622.7485	23.97991	15.89956
512.958	23.35977	10.29291	623.4263	23.96924	15.84621
513.6358	23.28958	10.2683	624.104	23.95653	15.78263
514.3135	23.21918	10.24396	624.7817	23.94188	15.70937
514.9912	23.14856	10.21989	625.4594	23.92539	15.62697
515.6689	23.07776	10.19609	626.1371	23.90719	15.53595
516.3466	23.00678	10.17256	626.8148	23.88737	15.43686
517.0244	22.93563	10.1493	627.4926	23.86605	15.33024
517.7021	22.86434	10.1263	628.1703	23.84332	15.21661
518.3798	22.79291	10.10357	628.848	23.81931	15.09653
519.0575	22.72137	10.0811	629.5257	23.79411	14.97053
519.7352	22.64972	10.0589	630.2034	23.76783	14.83913
520.413	22.57798	10.03696	630.8812	23.74058	14.70289
521.0907	22.50617	10.01528	631.5589	23.71247	14.56234
521.7684	22.4343	9.99387	632.2366	23.6836	14.41802
522.4461	22.36239	9.97271	632.9143	23.65409	14.27046
523.1238	22.29045	9.95181	633.592	23.62404	14.12021
523.8015	22.21849	9.93117	634.2698	23.59356	13.96779

524.4793	22.14654	9.91078	634.9475	23.56275	13.81375
525.157	22.0746	9.89065	635.6252	23.53173	13.65863
525.8347	22.00269	9.87078	636.3029	23.50059	13.50296
526.5124	21.93082	9.85116	636.9806	23.46946	13.34728
527.1901	21.85902	9.83179	637.6584	23.43843	13.19212
**wavelength**	ε_r	ε_i	**wavelength**	ε_r	ε_i
527.8679	21.78729	9.81268	638.3361	23.40761	13.03803
528.5456	21.71565	9.79381	639.0138	23.37711	12.88555
529.2233	21.64412	9.7752	639.6915	23.34704	12.7352
529.901	21.5727	9.75683	640.3692	23.31751	12.58753
530.5787	21.50142	9.73871	641.0469	23.28862	12.44308
531.2565	21.43029	9.72084	641.7247	23.26048	12.30237
531.9342	21.35932	9.70322	642.4024	23.23319	12.16596
532.6119	21.28853	9.68584	643.0801	23.20688	12.03437
533.2896	21.21794	9.6687	643.7578	23.18163	11.90815
533.9673	21.14755	9.65181	644.4355	23.15757	11.78784
534.6451	21.07739	9.63516	645.1133	23.13479	11.67396
535.3228	21.00746	9.61875	645.791	23.11341	11.56706
536.0005	20.93779	9.60258	646.4687	23.09353	11.46767
536.6782	20.86839	9.58665	647.1464	23.07527	11.37634
537.3559	20.79927	9.57096	647.8241	23.05872	11.29359
538.0336	20.73045	9.5555	648.5019	23.044	11.21998
538.7114	20.66194	9.54028	649.1796	23.03121	11.15603
539.3891	20.59375	9.5253	649.8573	23.02046	11.10228
540.0668	20.52591	9.51055	650.535	23.01185	11.05927
540.7445	20.45843	9.49603	651.2127	23.00551	11.02754
541.4222	20.39132	9.48175	651.8905	23.00152	11.00762
542.1	20.32459	9.4677	652.5682	23.00001	11.00006
542.7777	20.25827	9.45387	653.2459	23.01454	11.01066
543.4554	20.19237	9.44028	653.9236	23.06283	11.04593
544.1331	20.12689	9.42691	654.6013	23.14256	11.10388
544.8108	20.06187	9.41378	655.279	23.25128	11.18247
545.4886	19.9973	9.40087	655.9568	23.38651	11.27965
546.1663	19.93321	9.38818	656.6345	23.54579	11.39339
546.844	19.86961	9.37572	657.3122	23.72665	11.52163
547.5217	19.80651	9.36348	657.9899	23.92664	11.66235
548.1994	19.74394	9.35146	658.6676	24.14328	11.8135
548.8772	19.6819	9.33967	659.3454	24.37412	11.97303
549.5549	19.62042	9.3281	660.0231	24.61669	12.13891
550.2326	19.55949	9.31674	660.7008	24.86852	12.30909
550.9103	19.49915	9.30561	661.3785	25.12716	12.48153
551.588	19.4394	9.29469	662.0562	25.39013	12.65419
552.2657	19.38026	9.28399	662.734	25.65497	12.82502
552.9435	19.32175	9.2735	663.4117	25.91922	12.99199
553.6212	19.26388	9.26323	664.0894	26.18042	13.15305
554.2989	19.20666	9.25317	664.7671	26.43609	13.30616
554.9766	19.15011	9.24333	665.4448	26.68379	13.44928
**wavelength**	ε_r	ε_i	**wavelength**	ε_r	ε_i
555.6543	19.09424	9.23369	666.1226	26.92103	13.58036
556.3321	19.03908	9.22427	666.8003	27.14536	13.69737
557.0098	18.98462	9.21505	667.478	27.35431	13.79826
557.6875	18.9309	9.20605	668.1557	27.54542	13.88099
558.3652	18.87792	9.19725	668.8334	27.71623	13.94352
559.0429	18.82569	9.18866	669.5111	27.86426	13.98381
559.7207	18.77424	9.18028	670.1889	27.98706	13.99981
560.3984	18.72358	9.1721	670.8666	28.09343	13.99124
561.0761	18.67372	9.16412	671.5443	28.19977	13.9608
561.7538	18.62468	9.15635	672.222	28.30599	13.90988
562.4315	18.57647	9.14878	672.8997	28.41176	13.83983

563.1093	18.52911	9.14141	673.5775	28.51678	13.75201
563.787	18.48261	9.13424	674.2552	28.62072	13.64779
564.4647	18.43699	9.12727	674.9329	28.72328	13.52851
565.1424	18.39226	9.12049	675.6106	28.82413	13.39555
565.8201	18.34844	9.11391	676.2883	28.92297	13.25024
566.4978	18.30553	9.10753	676.9661	29.01948	13.09397
567.1756	18.26357	9.10135	677.6438	29.11334	12.92807
567.8533	18.22256	9.09536	678.3215	29.20425	12.75391
568.531	18.18251	9.08956	678.9992	29.29187	12.57285
569.2087	18.14344	9.08395	679.6769	29.37591	12.38624
569.8864	18.10537	9.07853	680.3547	29.45605	12.19545
570.5642	18.06831	9.07331	681.0324	29.53196	12.00183
571.2419	18.03228	9.06827	681.7101	29.60334	11.80674
571.9196	17.99729	9.06342	682.3878	29.66987	11.61154
572.5973	17.96335	9.05876	683.0655	29.73124	11.41758
573.275	17.93049	9.05428	683.7432	29.78713	11.22623
573.9528	17.8987	9.04999	684.421	29.83724	11.03884
574.6305	17.86802	9.04589	685.0987	29.88123	10.85678
575.3082	17.83846	9.04196	685.7764	29.9188	10.68139
575.9859	17.81002	9.03822	686.4541	29.94963	10.51404
576.6636	17.78272	9.03466	687.1318	29.97342	10.35608
577.3414	17.75659	9.03128	687.8096	29.98984	10.20888
578.0191	17.73163	9.02808	688.4873	29.99857	10.07379
578.6968	17.70786	9.02506	689.165	29.99993	9.95098
579.3745	17.6853	9.02222	689.8427	29.99919	9.83082
580.0522	17.66395	9.01955	690.5204	29.99762	9.71089
580.7299	17.64384	9.01706	691.1982	29.99517	9.59121
581.4077	17.62497	9.01474	691.8759	29.99184	9.47177
582.0854	17.60737	9.01259	692.5536	29.98758	9.3526
582.7631	17.59105	9.01062	693.2313	29.98237	9.2337
**wavelength**	$\boldsymbol{\varepsilon_r}$	$\boldsymbol{\varepsilon_i}$	**wavelength**	$\boldsymbol{\varepsilon_r}$	$\boldsymbol{\varepsilon_i}$
583.4408	17.57602	9.00882	693.909	29.97619	9.11509
584.1185	17.56229	9.00719	694.5868	29.969	8.99676
584.7963	17.54989	9.00572	695.2645	29.96079	8.87873
585.474	17.53883	9.00443	695.9422	29.95152	8.76102
586.1517	17.52913	9.0033	696.6199	29.94117	8.64363
586.8294	17.52078	9.00235	697.2976	29.9297	8.52656
587.5071	17.51382	9.00155	697.9753	29.9171	8.40984
588.1849	17.50826	9.00092	698.6531	29.90333	8.29347
588.8626	17.50411	9.00046	699.3308	29.88836	8.17745
589.5403	17.50139	9.00015	700.0085	29.87218	8.06181
590.218	17.50011	9.00001	700.6862	29.85475	7.94655
590.8957	17.5039	9.0042	701.3639	29.83604	7.83167
591.5735	17.52627	9.02829	702.0417	29.81603	7.7172
592.2512	17.56766	9.07286	702.7194	29.7947	7.60313
592.9289	17.62713	9.1369	703.3971	29.77201	7.48948
593.6066	17.70373	9.2194	704.0748	29.74793	7.37627
594.2843	17.79653	9.31934	704.7525	29.72244	7.26348
594.9621	17.90457	9.4357	705.4303	29.69552	7.15115
595.6398	18.02693	9.56746	706.108	29.66712	7.03928
596.3175	18.16264	9.71361	706.7857	29.63724	6.92787
596.9952	18.31077	9.87314	707.4634	29.60584	6.81694
597.6729	18.47038	10.04502	708.1411	29.57288	6.7065
598.3506	18.64052	10.22825	708.8189	29.53836	6.59655
599.0284	18.82025	10.4218	709.4966	29.50223	6.48711
599.7061	19.00862	10.62467	710.1743	29.46448	6.37819
600.3838	19.20469	10.83582	710.852	29.42507	6.2698
601.0615	19.40752	11.05425	711.5297	29.38397	6.16194
601.7392	19.61617	11.27895	712.2075	29.34117	6.05463

602.417	19.82968	11.50889	712.8852	29.29662	5.94787
603.0947	20.04713	11.74306	713.5629	29.25032	5.84168
603.7724	20.26755	11.98044	714.2406	29.20222	5.73606
604.4501	20.49002	12.22002	714.9183	29.1523	5.63103
605.1278	20.71359	12.46079	715.596	29.10053	5.52659
605.8056	20.9373	12.70171	716.2738	29.04689	5.42276
606.4833	21.16023	12.94179	716.9515	28.99135	5.31955
607.161	21.38143	13.18	717.6292	28.93388	5.21696
607.8387	21.59995	13.41533	718.3069	28.87446	5.115
608.5164	21.81484	13.64675	718.9846	28.81305	5.01368
609.1942	22.02518	13.87327	719.6624	28.74963	4.91302
609.8719	22.23	14.09385	720.3401	28.68418	4.81303

References

1. Schasfoort, R.B.M.; Tudos, A.J. *Handbook of Surface Plasmon Resonance*; The Royal Society of Chemistry: Cambridge, UK, **2008**; pp. 1–13.
2. Jackson, J. D.; *Classical Electrodynamics,* 3rd ed.; Wiley: New York, NY, USA, **1998**; pp. 1–135
3. Maier, S. A.; *Plasmonics: Fundamentals and Applications*. Springer: New York, NY, USA, **2007** pp. 49–74.
4. Liu, H.L.; Shen, C.C.; Su, S.H.; Hsu, C.L.; Li, M.Y.; Li, L.J. Optical properties of monolayer transition metal dichalcogenides probed by spectroscopic ellipsometry. *Appl. Phys. Lett.* **2014**, *105*, 201905.
